# Digital social contacts predict reduced loneliness in chronic depression: an exploratory longitudinal study

**DOI:** 10.1007/s00406-025-02179-6

**Published:** 2026-02-12

**Authors:** Johannes Wolf, Frank Padberg, Amelie Cronau, Katrin Wörz, Julia I. Kunz, Patrick Bach, Stephan Goerigk, Andrea Jobst, Fabienne Grosse-Wentrup, Matthias A. Reinhard

**Affiliations:** 1 Department of Psychiatry and Psychotherapy, LMU University Hospital, LMU Munich, Nussbaumstr. 7, 80336 Munich, Germany; 2 German Center for Mental Health (DZPG), Partner Site Munich-Augsburg, Munich, Germany; 3 Department of Addictive Behavior and Addiction Medicine, Central Institute of Mental Health, Mannheim, Germany; 4 German Center for Mental Health (DZPG), Partner Site Mannheim-Heidelberg- Ulm, Mannheim, Germany; 5 Charlotte Fresenius Hochschule, Munich, Germany

**Keywords:** Depression, Social media, Loneliness, Rejection sensitivity, Mental health

## Abstract

Social media use (SMU) is linked to loneliness and mental health. Yet its effects especially in severe and persistent mental disorders, such as chronic depression (CD), remain underinvestigated. Therefore, this observational longitudinal study investigated how SMU patterns relate to loneliness in CD and whether rejection sensitivity moderates these associations. A total of 124 individuals with CD completed measures of loneliness, rejection sensitivity, social networks, and SMU at baseline and after 10 weeks. Baseline correlation analyses identified significant associations of greater digital contacts and lower loneliness (*r* = − .33, *p* < .001). Moderated regression controlling for offline contacts revealed that more frequent digital contacts predicted reduced loneliness over time among participants with high baseline loneliness (β = − 0.23, *p* = .026). Preliminary data implies that this effect may further be moderated by rejection sensitivity. The absence of significant effects in the reverse prediction model supports directional inference. While necessitating replication, these findings suggest that contact-oriented SMU may buffer loneliness in CD beyond offline contacts and emphasize the relevance of addressing digital behavior in clinical care.

**Supplementary Information:**

The online version contains supplementary material available at 10.1007/s00406-025-02179-6.

## Introduction

Social media has transformed modern communication, reshaping how people initiate, maintain, and perceive social interactions. While its effects on mental health remain highly debated, its potential associations with loneliness have become a focus of investigation [1–4]. Loneliness can be defined as the subjective distress resulting from a perceived discrepancy between desired and actual social interactions [5]. As social media platforms mediate social interactions in modern communication, they may influence the experience of loneliness and therefore contribute to mental health distress.

Evidence regarding this influence is mixed. A growing body of research suggests that social media use (SMU) may alleviate loneliness through facilitating possibilities for social support and distraction from mental health problems [3, 6–8]. Other studies highlight risks such as social comparison resulting in a more negative self-view, perceived rejection, or cyberbullying, which can increase loneliness and even lead to suicidal behaviors [9–14]. These contradictory findings may partly stem from methodological limitations, such as the predominance of cross-sectional designs, lack of differentiated assessments, and a focus on general, non-clinical populations.

Even less longitudinal studies have been conducted, offering mixed results as well. One large longitudinal study suggested that SMU predicts increased mental health burden [15], while others indicate that vice versa declining well-being and loneliness may lead to greater SMU [1–3] or bidirectional associations were found [16]. These inconsistencies highlight the need for more nuanced approaches to better understand SMU patterns and investigate individual differences that moderate the association of SMU patterns with loneliness [1, 6, 12, 17, 18].

Emerging evidence points to rejection sensitivity as a potential moderator. Rejection sensitivity describes a tendency to fear rejection in social interactions, quickly interpret social cues as rejecting, and respond with heightened emotional distress [19]. Individuals with high rejection sensitivity have been shown to be more likely to interpret ambiguous or neutral (offline) social interactions as rejection, reinforcing loneliness and depressive symptoms in healthy as well as clinical groups [20]. Initial research suggests this trait may partially mediate responses to SMU [21] and our own research showed that rejection sensitivity is a key mechanism contributing to loneliness in chronic depression (CD) [20]. Yet, the role of rejection sensitivity in the relationship between SMU and loneliness remains underexplored.

Finally, studies increasingly recognize that the psychological effects of SMU may differ across diagnostic groups. While most research has focused on youth and general populations, relatively little is known about individuals with mental disorders [1, 2, 6, 22–25]. Limited evidence suggests frequent SMU in patients with affective disorders [24, 26]. Specifically, individuals with depression were reported to feel unhappier and experience more envy when comparing themselves to others on social media [18, 25, 27, 28]. One group for whom difficulties in social interactions and loneliness play a critical role in symptom severity and persistence, is CD. CD is also referred to as persistent depressive disorder according to DSM-5 [20, 29–33] and is conceptualized as a chronic mood disorder characterized by a longer, more enduring depressive course that is more disabling than episodic major depression [33]. In this group, to our knowledge no study has investigated the prevalence of digital social interactions, i.e., SMU, and its impact on mental health outcomes such as loneliness.

Therefore, this study aimed to investigate how SMU patterns are longitudinally associated with loneliness including rejection sensitivity as a potential moderator. Specifically, we investigated the following research questions.

1. What prevalence of SMU can be observed in individuals with CD?
2. How is SMU associated with loneliness in CD?
3. How do SMU and loneliness interplay over time?
4. Is rejection sensitivity a potential moderator of this association?

By focusing on distinct SMU patterns including digital contacts as well as conflicts in an extensively characterized clinical sample with CD, this study may help identify high-risk subgroups and SMU patterns to inform more personalized interventions that foster positive digital engagement while mitigating negative effects in vulnerable clinical populations with CD.

## Methods

### Design

This observational longitudinal study was conducted in a naturalistic setting within a 10-week inpatient treatment program with a focus on Cognitive Behavioral Analysis System of Psychotherapy as recommended and manualized to achieve the recommended dose of individual therapy sessions (20 individual sessions) as well as group therapy sessions [34, 35]. The treatment program did not focus on SMU or digital communication. All assessments were performed at admission (baseline) and after completion of the 10-week program. The study was carried out in accordance with the Declaration of Helsinki and approved by the Ethics Committee of the Faculty of Medicine at Ludwig Maximilian University (LMU) Munich (approval number #713–15; registered under DRKS00019821).

### Participants

Patients diagnosed with CD, i.e., persistent depressive disorder according to DSM-5, were recruited via convenience sampling at the Department of Psychiatry and Psychotherapy at the LMU University Hospital Munich, Germany. Patients were approached at admission to the program and all patients who participated provided written informed consent. Primary diagnosis of CD and comorbid psychiatric diagnoses were assigned according to DSM-5 criteria via structured clinical interviews (SCID-5-CV/ -PD, respectively). All measures were distributed in person.

### Measures

To assess loneliness, the study employed the German adaptation of the Revised UCLA Loneliness Scale (UCLA) to assess subjective experiences of loneliness and social isolation [36]. It comprises 20 items designed to measure the frequency and intensity of loneliness-related feelings, with responses rated on a five-point scale ranging from 1 (“not at all”) to 5 (“completely”). In addition, separate mean scores were derived for three specific subcomponents: feelings of loneliness, emotional isolation, and social isolation. Higher scores denote greater loneliness. The scale has been shown to exhibit high internal consistency (Cronbach’s alpha = 0.94) [37].

To assess SMU patterns, the study employed self-developed patient-rated items, which were designed to capture both SMU patterns and subjective experiences related to digital communication in everyday life over the past four weeks. Participants were first asked to report their average daily screen time in minutes across devices such as smartphones, personal computers, and tablets. Subsequently, they indicated how frequently they used various social media platforms - including *WhatsApp*,* Instagram*,* Facebook/Messenger*,* Telegram*,* Signal*, Email, SMS, and others - on a five-point Likert scale ranging from 0 (“never”) to 4 (“daily”). The same scale was used to assess how often participants communicated with different social groups, such as their partner, family members, friends, and colleagues, using one or more of these digital channels. In addition, the questionnaire included an item assessing how often participants experienced conflicts or unsatisfactory interactions in the context of SMU, also rated on the same five-point scale. Mean scores were calculated to assess SMU (mean use frequency of *Facebook*,* Instagram*,* Twitter*,* Signal*,* Telegram*,* Threema*,* and Whatsapp*), and digital contacts (mean contact frequency with partners, family, friends). While these items have not yet undergone formal psychometric validation, they were carefully constructed to reflect common forms of SMU including digital interactions relevant to individuals with CD. The questions can be found in the supplement.

To distinguish SMU effects from in-person social interactions, we controlled our analyses for offline social network characteristics. Social network characteristics were measured using the German version of the Social Network Index (SNI; [38]). This self-report instrument captures the number and diversity of social contacts across 12 relationship domains (e.g., spouse, parents, friends). For each domain, participants indicate how many people they are in contact with within the last 14 days. The SNI yields three subscale scores: network size (total number of contacts; SNI size), diversity (number of relationship domains with at least one contact; SNI diversity), and embeddedness (number of domains with high-contact persons; SNI embeddedness).

Rejection sensitivity was measured using the German version of the Rejection Sensitivity Questionnaire (RSQ) [19, 39]. The RSQ is a self-report instrument assessing the tendency to anxiously expect, readily perceive, and intensely react to interpersonal rejection. The short form used in this study consists of 9 hypothetical social scenarios (e.g., asking a friend for a favor), each rated on two dimensions: (1) the level of concern or anxiety about the possibility of rejection, and (2) the perceived likelihood of acceptance. Responses are given on a six-point Likert scale. A composite score is calculated by multiplying rejection anxiety and reversed acceptance expectancy for each item and averaging across all scenarios. This composite score was used for analyses. The RSQ has shown high internal consistency (Cronbach’s alpha = 0.88) [40].

Self-reported symptom severity was measured with the Beck Depression Inventory 2nd Version (BDI-II) [41]. BDI-II scores range from 0 to 63, with higher scores reflecting more severe depression. Cut-off values imply minimal (scores 9–13), mild (scores 14–19), moderate (scores 20–28), or severe (scores > 28) depression, with scores higher than 12 indicating clinically significant symptomatology [41]. The scale has been shown to have strong internal consistency (Cronbach’s alpha = 0.96) [41, 42].

### Statistical analyses

All statistical analyses were conducted using R version 4.3.0. The most commonly reported social media platforms, i.e., Instagram and Facebook, were reported separately in descriptive statistics. Due to skew in SMU variables, spearman correlations were conducted to explore associations between key baseline variables, including depression severity, loneliness, SMU, and rejection sensitivity. We used an explorative hierarchical approach, testing two-point-longitudinal associations only in those variables with the strongest, i.e. top one baseline association, and moderation effects of rejection sensitivity only in significant longitudinal models. To assess longitudinal associations, multiple regression models were calculated. We included baseline levels of the outcome variables (e.g., baseline loneliness when predicting loneliness after 10 weeks) as a moderator to account for potential floor effects and to assess differences based on symptom severity. All models were additionally controlled for baseline age, gender, SNI variables, and the baseline levels of the outcome variable. To examine the directionality of effects, we also ran a reverse prediction model, swapping outcome and predictor variables. In a final step, rejection sensitivity was added as a moderator to the significant regression models. All continuous predictors and outcomes were mean centered before analysis, and we probed significant interactions via simple‑slopes tests at − 1 SD, mean, and + 1 SD of the moderator. Moderation models were calculated in complete cases only. All statistical tests were two-tailed, and significance was set at *p* < .05. To account for multiple testing, regression models were interpreted after Bonferroni-correction.

## Results

### Sample characteristics and social media use at baseline

Complete data at baseline were available for *n* = 124 individuals diagnosed with CD (55.6% female; mean age = 39.6 years, SD = 12.2). More than half the sample (58.0%) had at least one comorbid mental disorder. Participants reported a mean of three prior inpatient and two prior outpatient treatments. Severe depressive symptoms were found, with mean BDI-II scores of 31.4 (SD = 10.7). Loneliness levels were elevated, with a mean UCLA loneliness score of 2.9 (SD = 0.8). Participants reported an average SNI size of 9.1 people (SD = 7.0). Regarding SMU, mean daily screen time was 189.7 min (SD = 144.8). Daily SMU was reported by 79.0% of participants. This seemed to be driven mainly by daily *WhatsApp* use (68.5% of participants), while daily use of more public-facing platforms such as *Instagram* (17.7% of participants) or *Facebook* (10.4% of participants) were reported less often. Digital contact records revealed that 41.1% of participants had daily interactions with their partners, 17.7% with their family, and 29.0% communicated daily with friends via social media. Regarding difficult interactions via SMU, 27.4% of participants reported experiencing frequent interpersonal conflicts during SMU several times a week or more. Elevated levels of rejection sensitivity were reported (mean RSQ score of 15.1, SD = 5.9). A detailed overview of sample characteristics is presented in Table 1.

**Table 1:** Sample characteristics

	Baseline sample (*n* = 124)
Demographics	
Age, M (SD)	39.6 (12.2)
Female sex, N (%)	69 (55.6)
Not in a relationship, N (%)	62 (50.0)
Education years, M (SD)	15.8 (3.9)
Clinical characteristics	
Outpatient treatments, M (SD)	2.0 (1.2)
Inpatient treatments, M (SD)	3.0 (2.3)
Comorbid mental disorder*, N (%)	72 (58.0)
BDI-II, M (SD)	31.4 (10.7)
UCLA loneliness, M (SD)	2.9 (0.8)
UCLA feelings of loneliness, M (SD)	2.9 (0.8)
UCLA emotional isolation, M (SD)	2.6 (1.0)
UCLA social isolation, M (SD)	3.2 (0.7)
SNI size, M (SD)	9.1 (7.0)
SNI diversity, M (SD)	3.5 (1.9)
SNI embeddedness, M (SD)	0.8 (0.9)
RSQ rejection sensitivity, M (SD)	15.1 (5.9)
Screen time and social media use	
Screen time in minutes per day, M (SD)	189.7 (144.8)
Daily SMU, N (%)	98 (79.0)
Daily *WhatsApp* use, N (%)	85 (68.5)
Daily *Instagram* use, N (%)	22 (17.7)
Daily *Facebook* use, N (%)	13 (10.4)
Daily digital contact *, N (%)	109 (87.9)
Conflicts several times a week or more, N (%)	34 (27.4)

Note. *BDI-II* Beck Depression Inventory, 2nd version; *UCLA* UCLA loneliness scale; *SNI* Social Network Index; *RSQ* Rejection Sensitivity Questionnaire; * with partner, family, friends; * at least one comorbid mental disorder

### Baseline associations of loneliness and clinical variables with social media use

Correlation analyses (Table 2) revealed a significant association between higher numbers of digital contacts and lower loneliness (UCLA total score: r_s_ = –0.33, *p* < .001), which was found for all UCLA subcomponents and all SNI indices (Table 2). Screen time was positively associated with SMU (r_s_ = 0.35, *p* < .001) and conflicts (r_s_ = 0.27, *p* = .003) and showed trend-level associations with greater loneliness (UCLA total score: r_s_ = 0.17, *p* = .066; emotional isolation: r_s_= 0.17, *p* = .055) and smaller values on the SNI (SNI size: r_s_ = –0.16, *p* = .079; embeddedness: r_s_ = –0.17, *p* = .058). SMU was associated with digital contacts (r_s_ = 0.31, *p* < .001), conflicts (r_s_ = 0.28, *p* = .002), and UCLA social isolation (r_s_ = − 0.20, *p* = .020). Correlations of SMU patterns with BDI-II or rejection sensitivity scores were not observed.

**Table 2:** Results of bivariate correlation analysis at baseline (Spearman)

	Screen time	Social Media Use	Digital contacts	Digital Conflicts
Social Media Use	0.35***			
Digital contacts	– 0.06	0.31***		
Digital conflicts	0.27**	0.28**	0.13	
UCLA loneliness total score	0.17(*)	– 0.17(*)	– 0.33***	0.15
UCLA feelings of loneliness	0.13	– 0.15	– 0.28**	0.17(*)
UCLA emotional isolation	0.17(*)	– 0.11	– 0.33***	0.19*
UCLA social isolation	0.09	– 0.20*	– 0.32***	– 0.00
SNI size	– 0.16(*)	0.07	0.29**	0.04
SNI diversity	– 0.08	0.07	0.36***	0.03
SNI embeddedness	– 0.17(*)	0.10	0.28**	0.05
RSQ rejection sensitivity	0.03	0.03	– 0.00	0.15
BDI-II	0.10	– 0.05	0.00	0.05

Note. *BDI-II* Beck Depression Inventory, 2nd version; *UCLA* UCLA loneliness scale; *SNI* Social Network Index; *RSQ* Rejection Sensitivity Questionnaire; most commonly used social media platforms are reported separately; trend-level associations (*p* < .10) are marked with (*); bold font was used to higlight significant or trend-level associations

### Longitudinal analyses predicting loneliness over time and vice versa

**Fig. 1:**
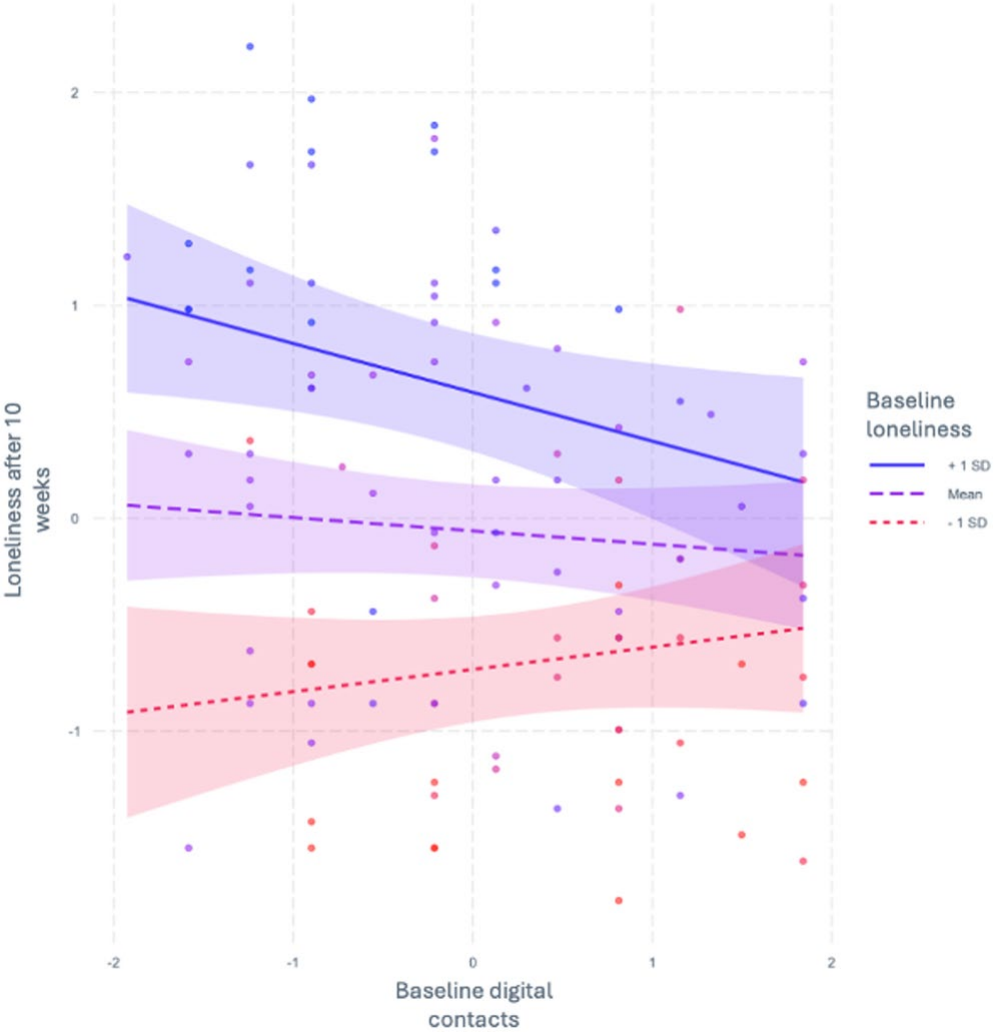
Slopes for model 1 predicting loneliness after 10 weeks by baseline digital contacts for different baseline loneliness levels. Note. Axis represent standard deviations of mean centered variables. Colors represent individuals with different baseline loneliness levels: blue = + 1 SD group; purple = mean group; red = -1 SD group

For longitudinal analysis, we focused on the strongest baseline association, i.e., the association of digital contacts with loneliness. In a first moderation model, we predicted loneliness after 10 weeks with baseline digital contacts for different levels of baseline loneliness controlling for age, gender, baseline SNI variables, and baseline loneliness (model 1). Model 1 (*n* = 102) accounted for 58.7% of the variance in follow‑up loneliness. Baseline loneliness was a significant predictor of later loneliness (β = 0.65, *p* < .001), whereas the main effect of baseline digital contacts was not significant (β = − 0.07, *p* = .310). However, the interaction between digital contacts and baseline loneliness was significant (β = − 0.16, *p* = .016). Simple‑slopes analyses showed that among participants with high baseline loneliness (+ 1 SD), greater baseline digital contact predicted significantly lower follow‑up loneliness (β = − 0.23, *p* = .026), whereas at mean or low baseline loneliness the corresponding slopes were non‑significant (β = − 0.06, *p* = .398; β = 0.10, *p* = .296, respectively). Slopes for model 1 are presented in Fig. 1.

We then calculated a vice-versa model, i.e., predicted digital contacts after 10 weeks by baseline loneliness for different levels of digital contacts at baseline (model 2). Model 2 accounted for 68.1% of the variance in follow‑up digital contacts, with a strong positive main effect of baseline digital contacts (β = 0.70, *p* < .001), but no significant effects of baseline loneliness (β = 0.04, *p* = .614) or its interaction with baseline digital contacts (β = 0.01, *p* = .863). Model statistics for models 1 and 2 are reported in Table 3.

**Table 3:** Model statistics for longitudinal analyses

	Model 1, *n* = 102 (predicting UCLA loneliness after 10 weeks)				Model 2, *n* = 74 (predicting digital contacts after 10 weeks)			
Baseline predictors	ß	SE	T (df = 93)	P value	ß	SE	T (df = 65)	P value
(Intercept)	0.01	0.10	0.06	0.944	– 0.01	0.11	– 0.07	0.941
Age	– 0.03	0.07	– 0.42	0.670	0.10	0.07	1.38	0.170
Gender (female)	0.01	0.14	0.09	0.927	– 0.09	0.15	– 0.59	0.556
SNI size	– 0.09	0.15	– 0.59	0.551	0.16	0.15	1.06	0.292
SNI diversity	– 0.12	0.11	– 1.11	0.266	– 0.01	0.12	– 0.07	0.943
SNI embeddedness	0.02	0.14	0.14	0.883	0.14	0.14	0.97	0.334
UCLA loneliness	**0.65**	**0.07**	**8.83**	< 0.001***	0.04	0.08	0.50	0.614
Digital contacts	– 0.07	0.07	– 1.02	0.310	**0.70**	**0.08**	**8.47**	< 0.001***
Digital contacts × UCLA loneliness (1)	**– 0.16**	**0.06**	**– 2.43**	0.016*	0.01	0.07	0.17	0.863
R^2^	0.58				0.68			

Note. (1) = for model 2 the interaction term was calculated vice versa; UCLA = UCLA loneliness scale; SNI = Social Network Index; bold font was used to higlight significant effects

### Rejection sensitivity as a moderator in the high loneliness group

Finally, we tested whether baseline rejection sensitivity moderated the significant longitudinal effect for the high-loneliness group (model 3, Table 4). In this small exploratory subsample (*n* = 14), our model explained a large proportion of variance (90.2%) and suggested a significant interaction between digital contacts and rejection sensitivity (β = 1.59, *p* = .012) with baseline digital contacts predicting reduced loneliness after 10 weeks at low (ß = − 3.34, *p* = .014) and average RSQ levels (ß = − 1.65, *p* = .028), but not at high RSQ levels (ß = 0.05, *p* = .891). Model statistics are reported in Table 4. However, given the small sample size in this analysis, statistical power is limited and the resulting estimates may be unstable. Accordingly, results should be interpreted with caution.

**Table 4:** Moderation model with RSQ-moderator predicting UCLA loneliness after 10 weeks, *n* = 14

Baseline predictors	ß	SE	t	*P* value
Intercept	– 1.97	1.02	– 1.92	0.127
Age	– 0.24	0.13	– 1.83	0.141
Gender (female)	– 0.27	0.35	– 0.78	0.477
SNI size	0.74	0.33	2.24	0.089
SNI diversity	**2.72**	**0.56**	**4.83**	0.008**
SNI embeddedness	– 1.14	0.43	– 2.66	0.056
UCLA loneliness	**2.02**	**0.61**	**3.32**	0.029*
Digital contacts	**– 1.65**	**0.49**	**– 3.39**	0.028*
RSQ total	0.66	0.33	2.01	0.114
Digital contacts × RSQ_total	**1.59**	**0.36**	**4.39**	0.012*
R^2^	0.90			

Note. *UCLA* UCLA loneliness scale; *SNI* Social Network Index; *RSQ* Rejection Sensitivity Questionnaire; bold font was used to higlight significant effects

## Discussion

This study investigated SMU patterns in individuals with CD and how they relate with loneliness over time, with an additional focus on the moderating role of rejection sensitivity. Addressing these questions in a well-characterized clinical sample using a longitudinal design contributes to filling a notable gap in existing literature, which has largely focused on non-clinical or youth populations and relied on cross-sectional data. The key findings can be summarized as follows: (1) SMU with a focus on digital contacts is a prevalent phenomenon in CD, (2) a higher number of digital contacts was associated with lower loneliness; (3) longitudinal analyses revealed that more frequent digital contacts predicted reduced loneliness over time among individuals with high baseline loneliness; and (4) preliminary data suggests that rejection sensitivity may moderate this effect, such that individuals with lower rejection sensitivity appeared to benefit most from digital contacts, while those with high rejection sensitivity may not experience these gains.

SMU was prevalent in CD. SMU in CD was characterized by frequent digital contacts with close social ties, i.e. friends, family, partners, and frequent messaging-based SMU via *WhatsApp.* This form of SMU was reported daily by the majority of participants (68.5%) and appears to reflect a relationship-oriented use of digital media. In contrast, frequently used public-facing platforms such as *Facebook or Instagram were* reported daily far less. The contrast between high daily use of messaging platforms and the limited use of visually-oriented, public-facing platforms may reflect key characteristics of CD [33], i.e., relationship-focused SMU may serve as a low-threshold avenue for maintaining social bonds, especially given the interpersonal withdrawal, social anxiety, and fear of rejection commonly observed in CD [20]. Conversely, the low use of platforms emphasizing self-presentation (e.g., *Instagram*) may indicate discomfort with social exposure as previously observed in individuals with depression [24, 25]. Sociodemographic factors may also contribute to these usage patterns. With a mean age of approximately 40 years, our sample was older than typical digital-native populations. Given that public-facing platforms have seen a rise in popularity mainly among younger cohorts in recent years, age-related trends may partly account for the observed preferences.

Digital contacts were robustly associated with lower loneliness across multiple dimensions (total loneliness, emotional isolation, and social isolation). This finding is consistent with previous evidence suggesting that certain types of SMU may be used to promote belongingness [1, 3]. Our longitudinal analyses further supported the notion that digital contacts can serve as a protective factor against loneliness in CD - but only under specific conditions. Among participants with high baseline loneliness, greater digital contact predicted significantly lower loneliness-levels after 10 weeks. Notably, this association was independent of age, gender, offline social network size and diversity, suggesting that digital contacts offer an additive benefit rather than merely reflecting broader social connectivity. In contrast, digital contacts had no effect in individuals with low or average baseline loneliness, suggesting that the protective role of digital contacts may be most relevant for those at elevated risk. The reverse prediction model - assessing whether baseline loneliness predicted digital contacts after 10 weeks - did not reveal significant effects. This asymmetry supports a directional interpretation that maintaining digital contacts may buffer against loneliness over time. These results resonate with broader findings from social media research in mostly non-clinical populations, although longitudinal data is scarce [1, 3]. For individuals with CD, who often struggle with maintaining in-person interactions due to anhedonia, avoidance, or interpersonal fears, digital contacts may offer an accessible means to fulfill social needs and reduce isolation. Additionally, our findings contribute to an ongoing debate about the meaningfulness of differentiating between “active” and “passive” SMU [43]. Including metrics of digital contacts may enhance the explanatory value of SMU categorization, suggesting a shift in focus from platform-based metrics to function-based behavioral indicators.

Our final analyses focused on the role of rejection sensitivity as a moderator in the longitudinal link between digital contacts and loneliness. Results suggest that this trait may significantly alter the association of digital contacts with loneliness over time, though the small subgroup size limits statistical power and warrants replication. The temporal association of digital contacts with reduced loneliness in individuals with high baseline loneliness only persisted in those with low to moderate rejection sensitivity. For participants with high rejection sensitivity, this benefit of digital contacts was not observed. This finding aligns with theoretical models of rejection sensitivity [19], as well as prior research regarding face-to-face [20] and digital interactions [21]. As such, the potential of digital contacts to reduce loneliness may be undermined by maladaptive interpretations of ambiguous social interactions, even in a text-based or asynchronous format. While warranting replication, our findings underscore the potential of addressing digital behavior in clinical care: therapeutic approaches could incorporate digital behavior as part of interpersonal interventions, encouraging structured, supportive engagement via messaging apps with existing social contacts. However, other patients - particularly those high in rejection sensitivity - may first need support in restructuring interpersonal expectations and interpretations before digital strategies can be effective.

Several limitations of this study should be noted. First, the observational design precludes definitive causal conclusions, although the directionality supported by reverse modeling and longitudinal data strengthens inference. Second, our SMU questionnaire, while clinically grounded, has not yet undergone psychometric validation. Future studies should validate this tool and incorporate objective measures of SMU (e.g., digital trace data) to enhance ecological validity. Third, the sample size for moderation analyses, particularly in the subgroup model, was limited. While our findings regarding rejection sensitivity are promising, they should be interpreted with caution and may only serve for generating hypotheses to be tested in future research, i.e., larger studies in clinical samples investigating rejection sensitivity as a moderator between SMU and loneliness. Fourth, the generalizability of our findings may be further constrained by the specific psychotherapeutic and inpatient context in which the data was collected. This may have confounded our findings, and future studies should replicate these findings in real-world contexts.

In conclusion, this study provides evidence that digital contacts may buffer loneliness in individuals with CD, particularly among those with elevated loneliness. These findings highlight the importance of considering not just *how much* social media is used, but *how* and *for what purpose* it is used. Digital contacts may offer a potentially meaningful, low-threshold avenue for maintaining social connection in vulnerable populations and may serve as an acceptable and potentially relevant adjunct in clinical care for CD. While evidence-based suggestions cannot yet be drawn from this observational study, clinicians should be aware of the pinnacle significance of SMU. Clinicians might consider routinely assessing patients’ SMU and informing them about the possible protective role of digital social contacts in relation to loneliness. In selected cases, encouraging patients to explore and reflect on their digital contacts within psychotherapy may provide additional support. Future work should replicate these findings in larger samples using smartphone tracking data and further investigate how digital engagement can be optimized to support recovery in affective disorders.

## Supplementary Information

Below is the link to the electronic supplementary material.

Supplementary Material 1

## Data Availability

The data that support the findings of this ongoing study are not openly available due to reasons of participant privacy and are available from the corresponding author upon reasonable request.
