# Supplementary material for: Digital social contacts predict reduced loneliness in chronic depression: an exploratory longitudinal study
Source: Eur Arch Psychiatry Clin Neurosci. 2026 Feb 12;276(4):1543–51. doi: 10.1007/s00406-025-02179-6 (PMC13233894; doi:10.1007/s00406-025-02179-6)

## Supplement

| **Supplementary Table 1. Moderation model with RSQ-moderator predicting UCLA loneliness after 10 weeks, n = 14** | | | | |
| --- | --- | --- | --- | --- |
| Baseline predictors | ß | SE | t | P value |
| Intercept | -1.97 | 1.02 | -1.92 | .127 |
| Age | -0.24 | 0.13 | -1.83 | .141 |
| Gender (female) | -0.27 | 0.35 | -0.78 | .477 |
| SNI size | 0.74 | 0.33 | 2.24 | .089 |
| SNI diversity | **2.72** | **0.56** | **4.83** | **.008**** |
| SNI embeddedness | -1.14 | 0.43 | -2.66 | .056 |
| UCLA loneliness | **2.02** | **0.61** | **3.32** | **.029*** |
| Digital contacts | **-1.65** | **0.49** | **-3.39** | **.028*** |
| RSQ total | 0.66 | 0.33 | 2.01 | .114 |
| Digital contacts × RSQ_total | **1.59** | **0.36** | **4.39** | **.012*** |
| R^2^ | .90 |  |  |  |
| Note. *UCLA = UCLA loneliness scale; SNI = Social Network Index; RSQ = Rejection Sensitivity Questionnaire.* | | | | |

**Supplementary Figure 1. Questionnaire to assess social media use and digital communication (German Version).**


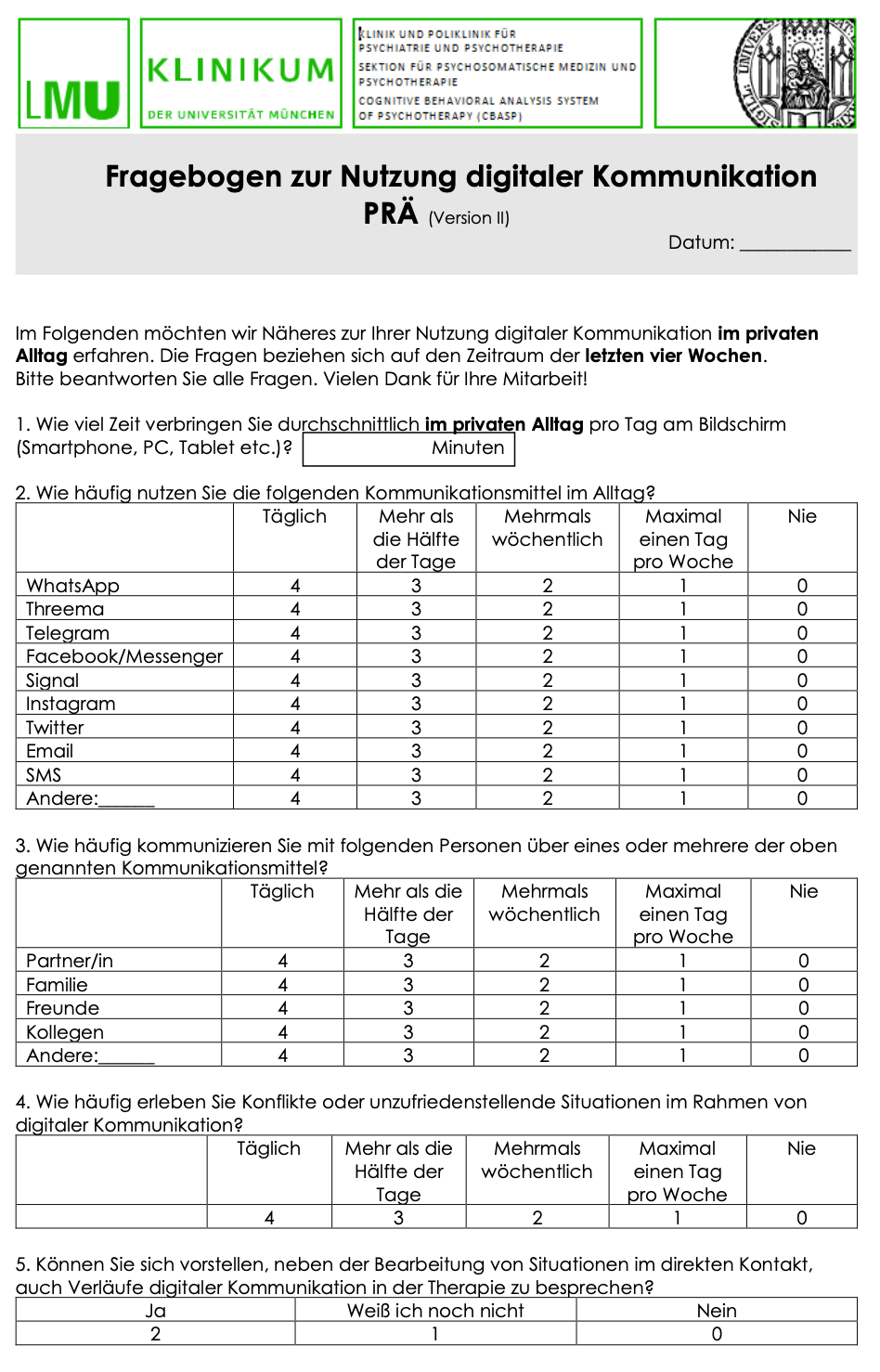

Supplement: Supplementary file 1 — Supplementary Material 1 [file 406_2025_2179_MOESM1_ESM.docx]
